# Supplementary material for: Enzyme-Mediated Quenching of the Pseudomonas Quinolone Signal (PQS): A Comparison between Naturally Occurring and Engineered PQS-Cleaving Dioxygenases
Source: Biomolecules. 2022 Jan 21;12(2):170. doi: 10.3390/biom12020170 (PMC8961568; doi:10.3390/biom12020170)
Supplement: Supplementary file 1 [file biomolecules-12-00170-s001.zip › biomolecules-1542744-supplementary.pdf]

Supplementary Material to:

Enzyme-mediated quenching of the *Pseudomonas* quinolone signal (PQS): a comparison between naturally occurring and engineered PQS-cleaving dioxygenases

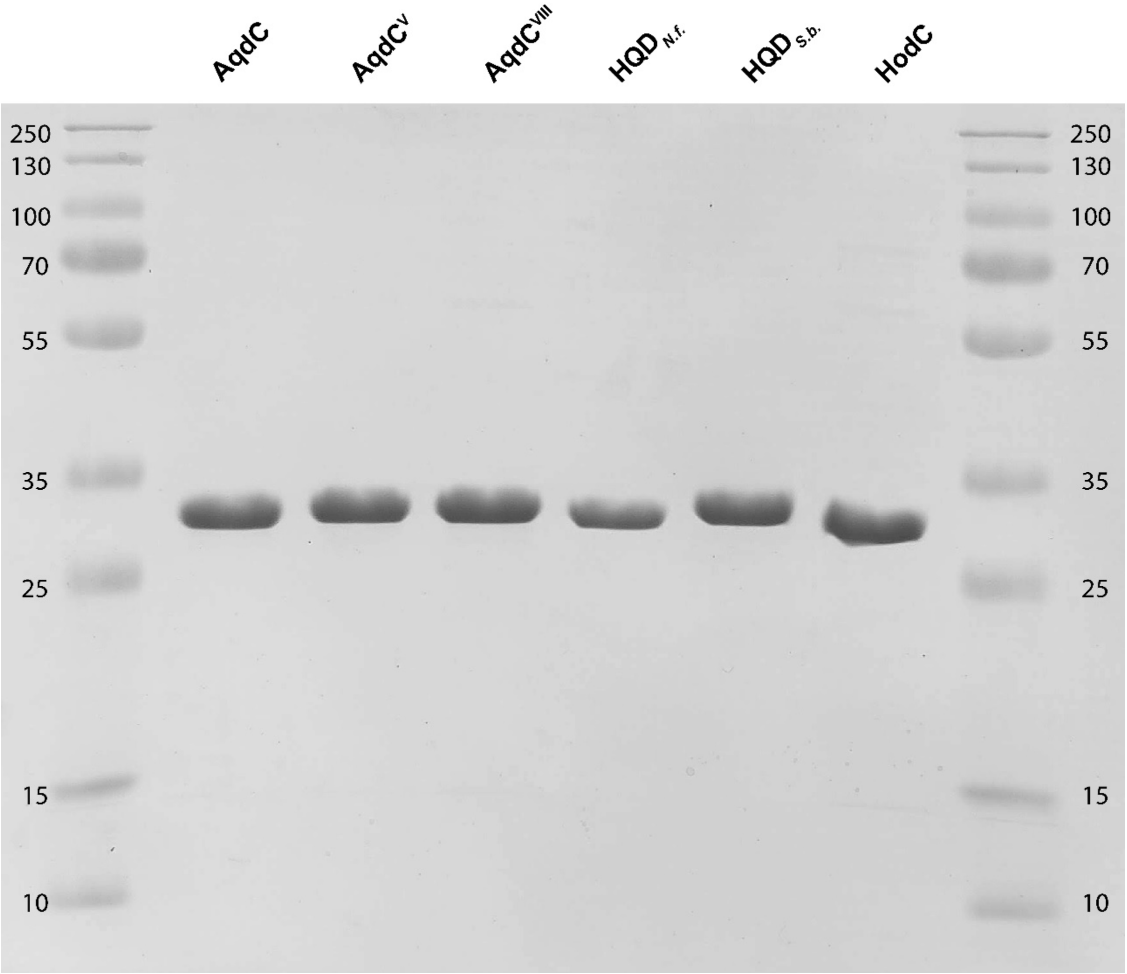

Figure S1. Purified HQD proteins. SDS-PAGE (12.5%) of recombinant proteins (4 µg) purified by affinity chromatography. Gels were stained with Coomassie Blue and PageRuler™ Plus Prestained Protein Ladder (Thermo Scientific) was used as a marker.

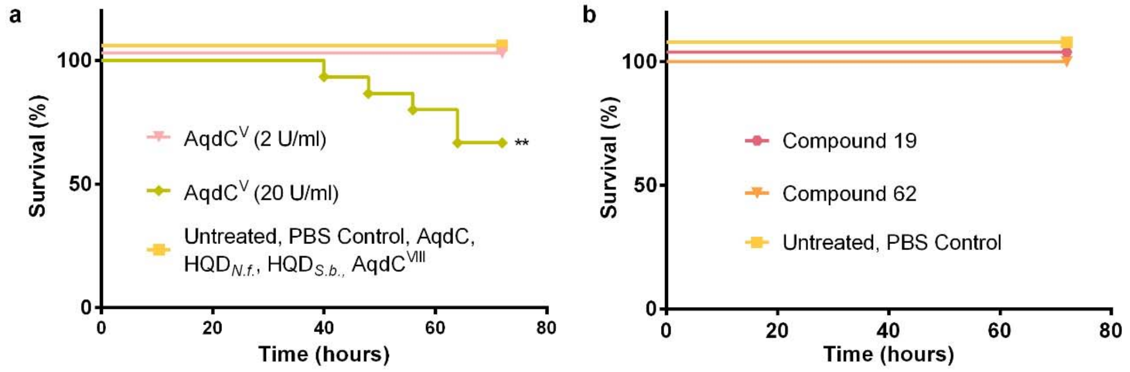

Figure S2. Effect of (a) PQS dioxygenases, and (b) PqsD inhibitors on survival of *G. mellonella* larvae. (a): Larvae were injected with 10 µL of HQD enzymes at a concentration of 20 U/mL ($HQD_{N.f.}$, $HQD_{S.b.}$, AqdC, $AqdC^{V}$ and $AqdC^{VIII}$) (yellow square and green diamond). The dioxygenase $AqdC^{V}$ was also injected at a concentration of 2 U/mL (pink triangle). (b): 10 mL of PqsD inhibitors 19 or 62 (100 µM) were injected. (a) and (b): Untreated larvae and larvae injected with the equivalent volume of PBS were included as control. After inoculation, larvae were incubated at 30°C in the dark and monitored every 8 hours over an 80h period. For clarity, results of the HQD dioxygenases treatments with the same outcome (a, yellow square) are not depicted individually.

Table S1. Kinetic parameters of HQDs for 2-heptyl-3-hydroxy-4(1*H*)-quinolone (PQS). Results are represented as mean ± SD.

Enzyme	Specific activity (U/mg)	k_{cat} (s^{-1})	K_M (µM)	k_{cat}/K_M	Reference
HodC	0.2	0.16	13.4	0.01	[15]
AqdC	60.2 ± 2.2	41.9 ± 1.1	5.8 ± 0.4	7.3 ± 0.6	[14]
$AqdC^{V}$	13.1 ± 0.2	15.9 ± 1.0	22.1 ± 4.1	0.7 ± 0.1	[19]
$AqdC^{VIII}$	43.5 ± 4.7	27.0 ± 2.8	3.7 ± 0.3	7.4 ± 0.1	[19]
$HQD_{N.f.}$	73.2 ± 2.8	43.9 ± 1.5	3.1 ± 0.4	14.1 ± 1.8	[14]
$HQD_{S.b.}$	34.1 ± 2.3	27.4 ± 2.3	2.7 ± 0.9	9.3 ± 1.1	[14]
